# Editorial: Women in psychiatry 2022: autism

**DOI:** 10.3389/fpsyt.2023.1208163

**Published:** 2023-05-15

**Authors:** Rita Barone, Costanza Colombi

**Affiliations:** ^1^Child Neuropsychiatry Unit, Department of Clinical and Experimental Medicine, University of Catania, Catania, Italy; ^2^Istituto di Ricerca e Cura a Carattere Scientifico (IRCCS) Fondazione Stella Maris, Pisa, Italy

**Keywords:** women—autism research, maternal-fetal heart rate interaction, autism spectrum disorder, gender differences, obesogenic factors, anorexia nervosa

We are proud to report on the second edition of the Frontiers Psychiatry research theme “*Women in Psychiatry 2022: Autism*.” This editorial will briefly discuss some of the highlights of the research presented. The overall male-to-female ratio (M:F) in ASD is estimated to be ~4:1, although there are differences in prevalence rates depending on some clinical variables, such as intellectual functioning, with a higher M:F in ASD subjects with normal cognitive levels (11:1), and chronological age, with the M:F decreasing to 2:1 in adulthood. It has been suggested that the higher male prevalence of ASD may be due to a diversity of gender-related genetic and environmental factors. In addition, it appears that girls with ASD may be more easily misdiagnosed with anxiety or depression than males. Two interesting studies dealing with gender differences in clinical symptoms of ASD were included in the present Research Topic. The research by Dellapiazza et al. aimed to investigate gender-related clinical differences in ASD patients without intellectual disability from the ELENA cohort (Longitudinal Study of Children with Autism). The study included 389 subjects (319 males) with ASD and normal cognitive level (IQ > 70), aged 2–11 years (mean age 6.3 ± 2.7 years). After ruling out gender differences in ASD symptom severity, adaptive functioning, IQ levels and sensory profiles, the study found that females were more impaired in social communication skills as measured by the Sensory Responsiveness Scale (SRS-2). In addition, the girls scored higher than boys on internalizing problems and anxiety problems on the CBCL scales. Overall, the study supports the occurrence of severe social impairment and higher levels of anxiety in females with ASD in childhood. The remarkable study by Paolizzi et al. investigated the occurrence of gender-related differences in ASD, focusing on interpersonal synchrony (IS) analyses. Fifty-one preschool children with ASD (male to female ratio 1:1) underwent a rigorous observational coding system to quantitatively examine the bidirectional interaction patterns displayed by the child and clinician during exchanges. The study shows that females with ASD engaged more efficiently than males which could affect the perceptions of others and ultimately lead to fewer referrals to services and delayed diagnosis for females with ASD. In addition, the study highlights the usefulness of IS analyses in disentangling more profound gender differences in social behavior in ASD.

The complex interplay between ASD and eating disorders was the subject of one creative study in this Research Topic (Li et al.). The analyses by Li et al. were based on clinicians' case notes of adult patients with ASD and anorexia nervosa to pragmatically inform clinicians' decision-making and treatment adaptations. Twenty individuals (80% female) with a formal diagnosis of autism or autistic characteristics and an eating disorder (ED) diagnosis (65% anorexia nervosa) were included in the study. The main challenges reported in this sample included communication and emotional difficulties, both of which interfered with therapeutic engagement. In addition, based on the authors' experience, it was particularly challenging to disentangle overlapping symptoms caused by autism, ED and common comorbidities such as OCD and anxiety. Notably, sensory disturbances and cognitive rigidity were prevalent in the fear of weight gain in people with autism and anorexia nervosa, thus altering the therapeutic approaches. In their research on the social lifestyle associated with obesity in children with autism, Kim and Kwon reported on the socio-ecological habits of 529 children with ASD aged 10–17 years from a nationwide survey. Sleep quality, parenting skills, parental health and participation in organized activities were significantly different between overweight (44%) and non-overweight children with ASD. The authors suggest that preventable social factors may contribute to the risk of obesity in children with ASD.

Finally, the work by Widatalla et al. used the valproate-induced mouse model to investigate the effects of the maternal environment on heart rate (HR) patterns during fetal development. Physiologically, the similarities between maternal and fetal RR intervals (RRIs) increase with embryonic days, suggesting that RRIs typically reflect fetal development. In this study, maternal and fetal heart rate correlations were analyzed using cross-correlation (CC) and magnitude-squared coherence (MSC) analyses. A disruption in the rhythm or regulation by which fetal and maternal HRs interact with each other was found in the valproate-treated mouse compared to the saline-treated mouse, suggesting an impairment in the interaction of fetal and maternal HRs in this ASD model.

In conclusion, this Research Topic of research articles highlights some current issues in ASD research and emphasizes the need to further investigate gender-related differences in ASD neurobehavioral phenotypes, as well as the effects of psychiatric co-morbidities (i.e., eating disorders) and modifiable lifestyles. A deeper understanding of maternal-fetal interactions in ASD experimental models is envisaged.

## Author contributions

RB and CC drafted and approved the final version of this editorial.

